# Research Progress on Downstream Mechanisms of Glucose Metabolic Reprogramming and Its Role in the Occurrence and Progression of Type 2 Diabetes Mellitus

**DOI:** 10.3390/biomedicines14071427

**Published:** 2026-06-24

**Authors:** Chan Wu, Maoying Wei, Aijing Li, Qingyi Zhu, Jingyi Guo, Anning Sun, Xin Gu, Yincheng Li, Yanbing Gong

**Affiliations:** Dongzhimen Hospital, Beijing University of Chinese Medicine, Beijing 100070, China; wuchan3461268442@163.com (C.W.); weimaoying2019@126.com (M.W.); liaijing129@163.com (A.L.); zqy020508@163.com (Q.Z.); jingyi_guobucm@163.com (J.G.); san13718818367@163.com (A.S.); 13322482107@163.com (X.G.); wuyasihuan@163.com (Y.L.)

**Keywords:** type 2 diabetes mellitus, glucose metabolic reprogramming, insulin resistance, mitochondria-associated endoplasmic reticulum membranes, oxidative stress

## Abstract

Type 2 diabetes mellitus (T2DM) is a highly prevalent and devastating chronic metabolic disease worldwide, with pathogenesis centrally characterized by insulin resistance and pancreatic β-cell dysfunction. Accumulating evidence has demonstrated that glucose metabolic reprogramming represents an adaptive metabolic shift from oxidative phosphorylation to aerobic glycolysis in cells in response to a hyperglycemic microenvironment. This shift acts as an upstream important event driving the initiation and progression of T2DM. This review summarizes the characteristics of glucose metabolic reprogramming in insulin-sensitive target organs under T2DM conditions, including the liver, skeletal muscle, adipose tissue and pancreatic β-cells. It also discusses four major downstream effector mechanisms: mitochondrial energy metabolism disturbance, augmented oxidative stress, disruption of mitochondria-associated endoplasmic reticulum membranes (MAMs) coupled with calcium homeostasis imbalance, and systemic inflammatory response. On this basis, we summarize the intervention strategies targeting the above signaling pathways, including antioxidant therapy, restoration of MAMs integrity and calcium homeostasis, systemic anti-inflammatory intervention, and multi-target regulatory effects of traditional Chinese medicine. Current studies indicate that early intervention in downstream stress events is induced by glucose metabolic reprogramming. This is particularly true for the preservation of MAMs’ integrity; restoration of calcium homeostasis; and inhibition of NLRP3 inflammasome activation, the latter of which is expected to block or delay the progression from prediabetes to clinical T2DM. Nevertheless, substantial gaps still remain in the understanding of the dynamic regulatory mechanisms of MAMs, tissue-specific therapeutic targets, and relevant clinical translational research. Future integration of multi-omics technologies will provide novel therapeutic strategies and theoretical foundations for the early prevention and treatment of T2DM.

## 1. Introduction

Diabetes mellitus (DM) is one of the most severe non-communicable chronic diseases, seriously endangering human health. According to the latest statistics released by the International Diabetes Federation (IDF), the global prevalence of DM reached approximately 589 million cases in 2024, and this figure is projected to rise to 853 million by 2050, with type 2 diabetes mellitus (T2DM) accounting for over 90% of all diabetic cases [1]. Emerging clinical evidence has demonstrated that patients with DM exhibit increased susceptibility to cancers of the digestive and reproductive systems. Additionally, these individuals face a higher risk of respiratory tract infections, cutaneous infections, and even systemic soft-tissue infections. Given its high mortality and disability rates, T2DM imposes a substantial economic burden on individual families and the entire healthcare system [2].

As a prevalent chronic metabolic disorder, T2DM has a complex pathogenesis predominantly characterized by disrupted glucose homeostasis and insulin resistance. The two main core pathological mechanisms are the impaired insulin secretion capacity of pancreatic β-cells, which disrupts systemic glucose regulation, and the diminished insulin responsiveness of insulin-sensitive target organs, which collectively exacerbate disease progression [3]. Abnormal metabolic signal transduction invariably impairs the regulatory function of this axis. Meanwhile, sustained glucolipid metabolic disturbance triggers glucotoxicity and lipotoxicity, which act as central pathological stimuli to initiate glucose metabolic reprogramming in insulin-sensitive organs, including the liver, skeletal muscle, adipose tissue, and pancreatic β-cells. Such metabolic alterations occur in multiple biological processes, such as glucose uptake, glycolysis, and oxidative phosphorylation. Mechanistically, dysregulated glucose metabolism disrupts cellular energy homeostasis and further provokes a cascade of downstream pathological abnormalities, including chronic inflammatory responses, excessive oxidative stress, mitochondrial dysfunction, activated endoplasmic reticulum stress, and aberrant mitochondria-associated endoplasmic reticulum membranes (MAMs). These detrimental pathological changes synergistically aggravate insulin resistance and deteriorate pancreatic β-cell function, ultimately accelerating the onset and progression of T2DM.

The concept of metabolic reprogramming was initially proposed in cancer therapy. It refers to the metabolic rewiring of signaling pathways in tumor cells, which remodels the flux and distribution of nutrients to satisfy energy demands and anabolic requirements, thereby sustaining tumor cell survival and proliferation [4]. Emerging evidence has revealed that cellular metabolic pathways and metabolites undergo adaptive modulation under pathological conditions of cardiovascular, central nervous system, renal, and respiratory diseases [5,6,7]. This metabolic remodeling is characterized by impaired mitochondrial biogenesis, enhanced glycolysis, and dysregulated lipid and amino acid metabolism, which has been recognized as a contributor facilitating the development and progression of diabetes.

## 2. Glucose Metabolic Reprogramming: A Core Metabolic Signature of T2DM

Dysregulated feedback modulation between insulin secretion and peripheral insulin action leads to persistent hyperglycemia and continuously promotes the pathophysiological progression of T2DM. On the one hand, the progressive deterioration of pancreatic β-cell function causes insulin secretory defects, impairing the intrinsic capacity for blood glucose homeostasis. On the other hand, insulin resistance markedly inhibits glucose uptake and utilization in the liver, skeletal muscle, and adipose tissue, while concurrently enhancing hepatic gluconeogenesis, thereby further exacerbating metabolic dysregulation. This review summarizes the regulatory roles of organ-specific mediators derived from major insulin-sensitive tissues (e.g., liver, skeletal muscle, and adipose tissue), as well as insulin-secreting regulatory organs including the pancreas and intestine, in the maintenance of glucose metabolic homeostasis.

Diabetes mellitus is tightly correlated with the progression of multiple liver diseases, covering a spectrum of pathological stages ranging from fatty liver and non-alcoholic fatty liver disease (NAFLD) to hepatic fibrosis, cirrhosis, and even hepatocellular carcinoma. Under physiological conditions, insulin effectively suppresses hepatic gluconeogenesis and modulates lipolysis. Nevertheless, in the diabetic pathological microenvironment, hepatic insulin sensitivity is markedly diminished, accompanied by disrupted insulin signaling. Such abnormalities contribute to excessive hepatic gluconeogenesis and sustained hyperglycemia, alongside dysregulated lipolysis that triggers excessive lipid accumulation in hepatocytes. Aberrant intracellular lipid deposition further induces oxidative stress, thereby causing hepatocellular dysfunction and injury. Damaged hepatocytes trigger the activation of Kupffer cells, which secrete abundant pro-inflammatory cytokines and initiate persistent low-grade chronic inflammation. In response to hepatic tissue damage, hepatic stellate cells synthesize and accumulate excessive extracellular matrix, ultimately resulting in progressive liver fibrosis and irreversible hepatic impairment [8].

Skeletal muscle constitutes one of the predominant insulin-sensitive tissues in the human body and serves as the central effector tissue mediating insulin-dependent glucose uptake; accordingly, skeletal muscle health directly determines systemic glucose homeostasis [9]. As critical myogenic progenitor cells responsible for the maintenance of muscle tissue integrity, skeletal muscle satellite cells exert essential functions in muscle fiber repair, remodeling, and the modulation of tissue plasticity [10,11]. Accumulating evidence has validated that diabetic conditions markedly impair the abundance and functional activity of skeletal muscle satellite cells, thereby triggering the onset and progression of diabetic myopathy and accelerating the deterioration rate of diabetic comorbidities [12].

Adipose tissue acts as a central organ for systemic energy storage. Subcutaneous adipose tissue is abundant in mitochondria with thermogenic capacity [13,14], and its biological function is tightly linked to the improvement of metabolic homeostasis and insulin sensitivity. Once energy intake exceeds the storage threshold of subcutaneous adipose tissue, excessive lipids undergo ectopic deposition in multiple tissues and organs, including the liver, vascular walls, and skeletal muscle. This process ultimately facilitates the initiation and progression of insulin resistance [15,16,17]. Beyond energy storage, adipose tissue also exerts crucial endocrine functions and secretes adiponectin, diverse cytokines, and bioactive lipid molecules. Both adipose tissue and tissue-infiltrating macrophages can robustly release pro-inflammatory mediators, such as interleukin-6, tumor necrosis factor-α, resistin, visfatin, retinol-binding protein 4, and leptin [18,19]. The expression levels of these factors are positively correlated with systemic fat mass, which aggravates insulin resistance and accelerates T2DM pathological progression by mediating persistent systemic low-grade chronic inflammation. Collectively, T2DM is characterized by systemic glucose metabolic dysregulation. Specific glucose metabolic reprogramming occurs in key target organs, including the liver, skeletal muscle, adipose tissue, and pancreatic β-cells, which jointly constitute the core metabolic phenotype driving disease progression.

## 3. Core Downstream Mechanisms of Glucose Metabolic Reprogramming

### 3.1. Imbalanced Energy Metabolism

**Figure 1:**
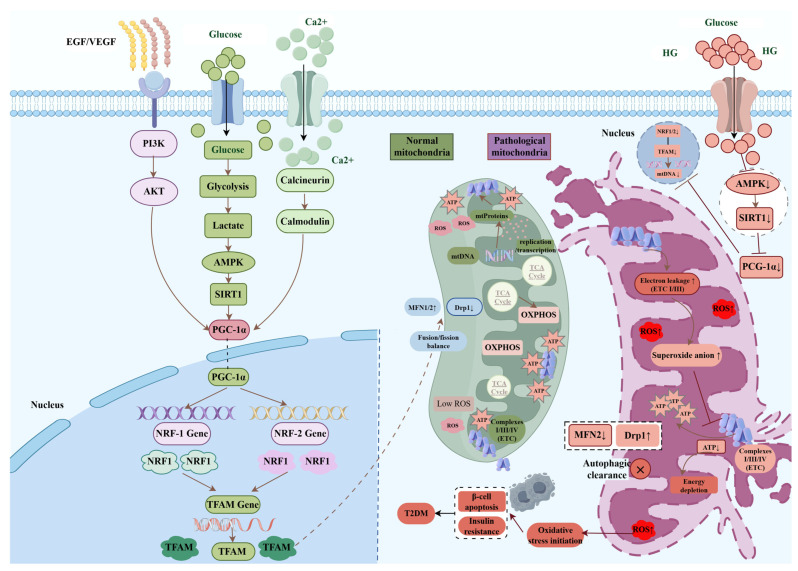
Imbalanced energy metabolism. EGF/VEGF—epidermal growth factor/vascular endothelial growth factor; PI3K—phosphatidylinositol 3-kinase; AKT—protein kinase B; Ca^2+^—calcium ion; calcineurin—serine/threonine protein phosphatase 2B; calmodulin—calcium-modulated protein; HG—high glucose; AMPK—AMP-activated protein kinase; SIRT1—sirtuin 1; PGC-1α—peroxisome proliferator-activated receptor gamma coactivator 1-alpha; NRF1/2—nuclear respiratory factor 1/2; TFAM—mitochondrial transcription factor A; mtDNA—mitochondrial DNA; ETC—electron transport chain; OXPHOS—oxidative phosphorylation; TCA cycle—tricarboxylic acid cycle; MFN1/2—mitofusin 1/2; Drp1—dynamin-related protein 1; ROS—reactive oxygen species; ATP—adenosine triphosphate; β-cell—pancreatic β cell.

Glucose metabolic reprogramming initially manifests as decreased mitochondrial oxidative phosphorylation efficiency and disrupted mitochondrial dynamics [20]. The core of mitochondrial dysfunction lies in the systematic imbalance of nutrient signal transduction, energy production, and redox metabolism, ultimately leading to inadequate adenosine triphosphate (ATP) synthesis and subsequent metabolic crisis [21,22]. In response to systemic metabolic disorders, mitochondria undergo a spectrum of pathological alterations, including aberrant substrate oxidation, impaired calcium buffering, disrupted iron transport, morphological dynamic remodeling, reactive oxygen species (ROS) burst, and activation of cellular apoptotic signaling cascades [23,24]. These pathological changes are intuitively illustrated in Figure 1.

Mitochondrial biogenesis and dynamic regulation serve as two essential pillars for the maintenance of mitochondrial functional homeostasis. Mitochondria originate from healthy eukaryotic cells and expand their quantity through the fission of fully mature and genetically replicated mitochondrial organelles. As a key regulator of mitochondrial biogenesis, peroxisome proliferator-activated receptor gamma coactivator-1α (PGC-1α) responds to the stimulation of tyrosine kinase receptors and natriuretic peptide receptors as well as nitric oxide. Subsequent post-translational modifications include sirtuin 1 (SIRT1)-mediated deacetylation and activation triggered by alterations in the adenosine monophosphate/adenosine triphosphate (AMP/ATP) ratio via adenosine monophosphate-activated protein kinase (AMPK) [25]. Combined with the modulation of calmodulin signaling, PGC-1α sequentially activates downstream nuclear transcription factors, such as nuclear respiratory factor 1/2 (NRF1/2) and estrogen-related receptor-α (ERR-α), thereby initiating de novo mitochondrial biogenesis [26,27]. Meanwhile, AMPK and SIRT1 form a metabolic sensing circuit that further promotes PGC-1α phosphorylation and enhances its transcriptional activity [28,29]. At the dynamic regulatory level, the physiological balance between mitochondrial fusion and fission is precisely modulated by multiple GTPases. Mitofusin 1/2 (MFN1/2) mediates the fusion of the mitochondrial inner and outer membranes [30], while optic atrophy 1 (OPA1) anchors to the inner mitochondrial membrane to facilitate inner membrane remodeling [31]. As a crucial GTPase fission mediator, dynamin-related protein 1 (DRP1) translocates from the cytoplasm to the outer mitochondrial membrane to drive mitochondrial fission, which guarantees the selective autophagic clearance of damaged mitochondria and maintains cellular homeostasis under stress conditions [32].

Under high-glucose conditions, the mitochondrial regulatory network undergoes two pathological shifts. Decreased PGC-1α expression and reduced MFN2 levels impair mitochondrial synthesis, while aberrant Drp1 activation enhances mitochondrial fission. These changes lead to the accumulation of fragmented mitochondria. Concurrently, electron leakage from the respiratory chain complex is significantly increased, resulting in excessive superoxide anion production. These alterations cause insufficient ATP synthesis and exacerbate oxidative stress [33]. Clinical studies have shown that the glucolipid metabolic disorder microenvironment in T2DM patients significantly inhibits the AMPK-SIRT1-PGC-1α axis. This suppresses lipid oxidation and aggravating lipid-induced insulin resistance [34,35]. In animal models, high-glucose conditions induce pathological mitochondrial dysfunction through dual mechanisms—epigenetic modifications such as H4K12 lactylation and post-translational modifications including Sirt1-mediated deacetylation impairment [36,37]. In vitro experiments further demonstrated that in high-glucose-treated 3T3-L1 adipocytes, the levels of mitochondrial protein complexes III and IV are reduced. Moreover, high glucose decreases PGC-1α expression by inhibiting AMPK phosphorylation, thereby accelerating mitochondrial fragmentation [38].

Although mitochondrial fragmentation is commonly observed in T2DM, whether it is a cause or a consequence remains subject to debate. Genetic studies suggest that polymorphisms in fusion-related genes such as MFN2 and OPA1 are associated with increased T2DM risk, supporting a primary pathogenic role for abnormal mitochondrial dynamics [39]. However, most intervention studies have shown that short-term high-glucose treatment is sufficient to induce mitochondrial fragmentation, suggesting it may be a secondary change [40]. A more plausible explanation is that these factors form a vicious cycle: initial metabolic disturbances cause mild mitochondrial fragmentation, leading to decreased insulin sensitivity, and the consequent worsening of metabolic disturbances further promotes fragmentation.

### 3.2. Exacerbated Oxidative Stress

**Figure 2:**
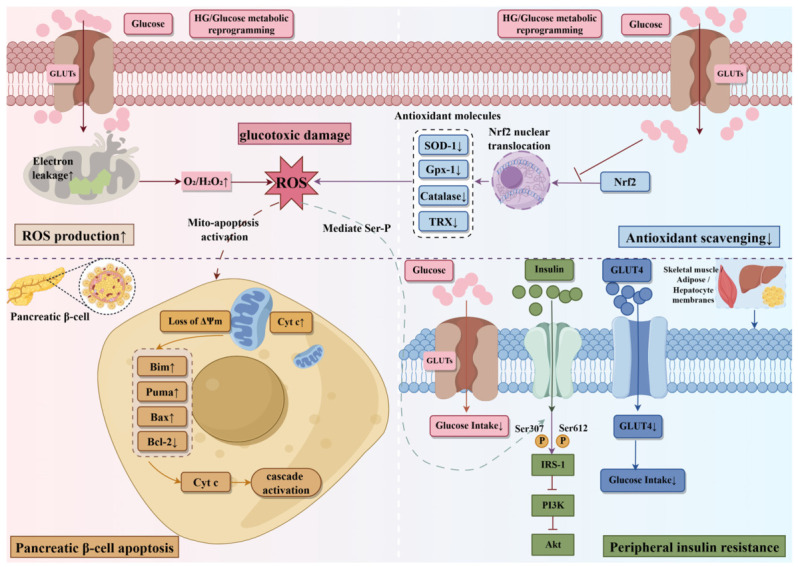
Exacerbated oxidative stress. GLUTs—glucose transporters; HG—high glucose; ROS—reactive oxygen species; O_2_/H_2_O_2_—oxygen/hydrogen peroxide; SOD-1—superoxide dismutase 1; Gpx-1—glutathione peroxidase 1; TRX—thioredoxin; Nrf2—nuclear factor erythroid 2-related factor 2; ΔΨm—mitochondrial membrane potential; Cyt c—cytochrome c; Bim—Bcl-2-interacting mediator of cell death; Puma—p53 upregulated modulator of apoptosis; Bax—Bcl-2-associated X protein; Bcl-2—B-cell lymphoma 2; IRS-1—insulin receptor substrate 1; PI3K—phosphatidylinositol 3-kinase; Akt—protein kinase B; GLUT4—glucose transporter 4.

Oxidative stress is a key mediator of glucotoxicity-induced damage. It arises from an imbalance between excessive mitochondrial ROS production and depletion of the endogenous antioxidant defense system (Figure 2). In T2DM, hyperglycemia is closely associated with elevated oxidative stress. Reactive species such as hydrogen peroxide (H_2_O_2_) and superoxide (O_2_^−^) have been identified as key drivers in the pathogenesis of diabetic complications [41,42]. Acute hyperglycemia promotes glucose entry into endothelial cells via glucose transporters (GLUTs). This induces mitochondrial dysfunction through the polyol pathway and causes uncoupling of endothelial nitric oxide synthase (eNOS), both of which increase ROS production [43,44]. Notably, pancreatic β-cells have intrinsically low expression of antioxidant enzymes such as SOD-1, Gpx-1, and catalase, making them highly sensitive to oxidative stress. Thus, under high-glucose conditions, increased ROS production coexists with relative depletion of the antioxidant enzyme system, forming the metabolic basis of β-cell injury [45,46]. In insulin target tissues, ROS directly contribute to insulin resistance by oxidative modification of key molecules in the insulin signaling pathway. Mechanistically, ROS induce phosphorylation of insulin receptor substrate-1 (IRS-1) at Ser307/Ser612, which inhibits downstream PI3K/Akt signaling and leads to peripheral insulin resistance [47].

In mammalian cells, the extrinsic death receptor pathway and the intrinsic mitochondrial (Bcl-2-regulated) pathway are the primary mechanisms of ROS-mediated β-cell injury. Excessive ROS production regulates the BAG3/Nrf2/HO-1 signaling axis, exacerbating oxidative stress and triggering pancreatic β-cell apoptosis. This process aggravates complications in diabetic patients [48]. Further studies have shown that ROS induce β-cell death mainly through the mitochondrial pathway by promoting loss of mitochondrial membrane potential, cytochrome c release, and caspase cascade activation. Intervention studies have demonstrated that overexpression of the pro-survival protein Bcl-2, or deficiency in the BH3-only proteins Bim and Puma and their downstream apoptotic effector Bax, significantly reduces high-glucose- or ribose-induced islet cell death [49,50]. Clinical and animal studies have also shown significantly increased mRNA expression of pro-apoptotic BH3-only molecules in islets of T2DM patients and mice, suggesting that hyperglycemia activates the Bim/Puma pathway to induce pancreatic β-cell apoptosis by integrating endoplasmic reticulum stress and mitochondrial oxidative stress [51]. Importantly, the damaging effects of oxidative stress are not limited to mitochondria. Acting as a signal amplification hub, oxidative stress also affects the endoplasmic reticulum and MAMs, laying the foundation for subsequent dysfunction in organelle crosstalk.

### 3.3. Organelle Crosstalk

**Figure 3:**
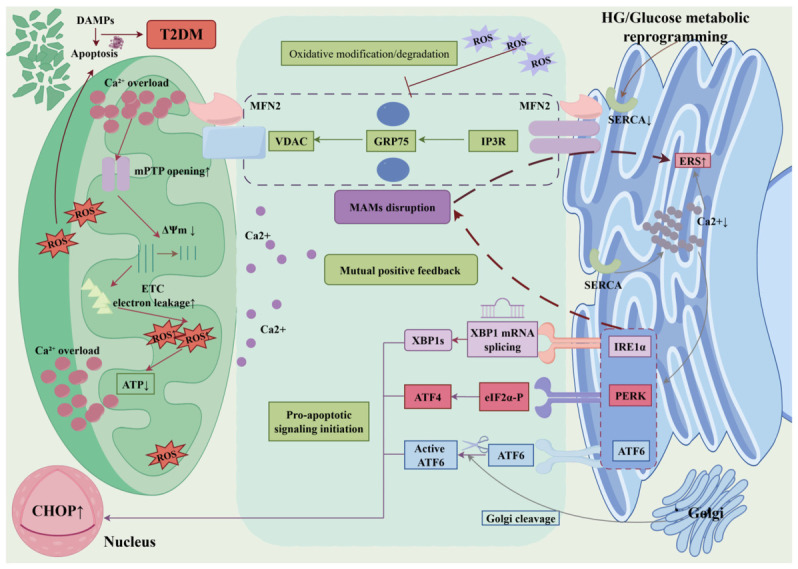
Organelle crosstalk. DAMPs—damage-associated molecular patterns; T2DM—type 2 diabetes mellitus; Ca^2+^—calcium ion; mPTP—mitochondrial permeability transition pore; ΔΨm—mitochondrial membrane potential; ETC—electron transport chain; ATP—adenosine triphosphate; ROS—reactive oxygen species; MFN2—mitofusin 2; VDAC—voltage-dependent anion channel; GRP75—glucose-regulated protein 75; IP3R—inositol 1,4,5-trisphosphate receptor; MAMs—mitochondria-associated endoplasmic reticulum membranes; SERCA—sarco/endoplasmic reticulum calcium ATPase; ERS—endoplasmic reticulum stress; IRE1α—inositol-requiring enzyme 1α; XBP1—X-box binding protein 1; XBP1s—spliced X-box binding protein 1; PERK—protein kinase R-like endoplasmic reticulum kinase; eIF2α-P—phosphorylated eukaryotic initiation factor 2α; ATF4—activating transcription factor 4; ATF6—activating transcription factor 6; CHOP—C/EBP homologous protein.

MAMs are specialized contact regions between mitochondria and the endoplasmic reticulum with a width of approximately 10–25 nanometers [52]. Their core functions include calcium exchange, mitophagy, endoplasmic reticulum stress, and glycolipid metabolism [52,53,54]. MAMs contain numerous resident proteins, such as MFN2, Grp75, PERK, and IP3R [55,56,57]. Dysregulation of these proteins contributes to the development and progression of diseases such as T2DM (Figure 3).

Under normal conditions, MAMs facilitate calcium transfer from the ER to mitochondria, thereby maintaining mitochondrial calcium homeostasis. Most enzymes involved in glycolipid biosynthesis are located on the ER membrane, with a minority on the mitochondrial membrane [58,59]. Thus, different lipid intermediates require MAMs for transfer to complete biosynthetic pathways. Additionally, MAMs can initiate the unfolded protein response to manage ER stress, promoting cell survival [60]. Under pathological conditions in T2DM, oxidative stress inhibits SERCA pump activity [61], leading to ER calcium depletion. This depletion activates three ER stress pathways: PERK, IRE1, and ATF6 [62]. These signals are mediated by MAM-associated proteins such as MFN2, IP3R, and GRP75, causing local mitochondrial calcium overload [63,64]. Consequently, the mitochondrial permeability transition pore (mPTP) opens, leading to membrane potential collapse, ATP synthesis arrest, and ROS production [65]. Notably, a bidirectional positive feedback loop exists between ER stress and MAM disruption. MAM dysfunction worsens ER calcium homeostasis imbalance. Conversely, ER stress degrades MAM-associated proteins through an IRE1α-dependent pathway, further compromising MAM integrity.

Clinical evidence indicates that presenilin (PS1/PS2) mutations associated with familial Alzheimer’s disease not only alter protein processing but also directly impair SERCA pump function and ER calcium leak dynamics. These changes ultimately activate the pro-apoptotic PERK-CHOP signaling axis [66]. This mechanism is commonly observed in T2DM-related organ damage. In podocytes, high glucose weakens the interaction between Mfn2 and PERK [67]. In hepatocytes, Grp75 knockdown inhibits IP3R-VDAC and IP3R-Grp75 interactions, impairing ER-mitochondria calcium transfer [68]. In pancreatic β-cells, ER stress activates the PERK-ATF4-CHOP pathway, upregulates TRB3 (a negative regulator of Akt signaling), and promotes apoptosis [69].

Although MAM disruption is widely recognized to be involved in T2DM pathogenesis, the direction of MAM function in insulin resistance remains subject to debate. Tubbs et al. demonstrated that MAM integrity is required for maintaining hepatic insulin signaling, and MAM disruption exacerbates insulin resistance [68,70]. However, other evidence suggests that excessive MAM enhancement is also detrimental. An abnormal increase in MAMs can lead to mitochondrial calcium overload, triggering mPTP opening and apoptosis [71]. This bidirectional phenomenon, in which both insufficient and excessive MAM function are harmful, suggests that MAM function may follow a “U-shaped curve,” and maintaining dynamic balance is key to therapeutic intervention.

### 3.4. Systemic Inflammation

**Figure 4:**
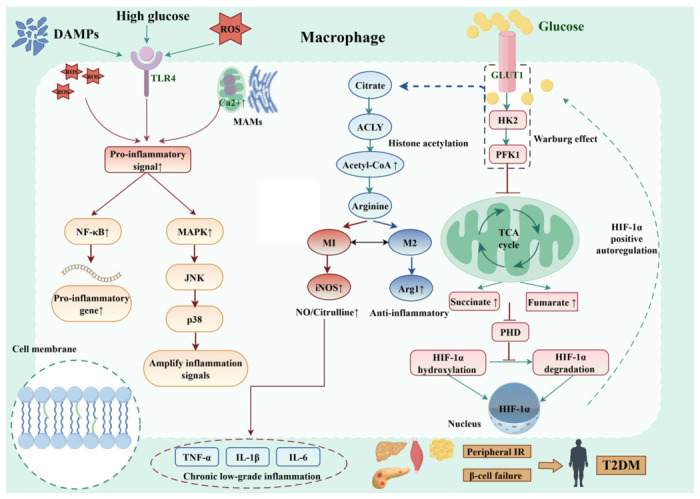
Systemic inflammation. DAMPs—damage-associated molecular patterns; ROS—reactive oxygen species; TLR4—toll-like receptor 4; MAMs—mitochondria-associated endoplasmic reticulum membranes; Ca^2+^—calcium ion; NF-κB—nuclear factor kappa-light-chain-enhancer of activated B cells; MAPK—mitogen-activated protein kinase; JNK—c-Jun N-terminal kinase; TNF-α—tumor necrosis factor-alpha; IL-1β—interleukin-1β; IL-6—interleukin-6; GLUT1—glucose transporter 1; HK2—hexokinase 2; PFK1—phosphofructokinase 1; ACLY—ATP-citrate lyase; Acetyl-CoA—acetyl coenzyme A; TCA cycle—tricarboxylic acid cycle; PHD—prolyl hydroxylase; HIF-1α—hypoxia-inducible factor 1-alpha; iNOS—inducible nitric oxide synthase; Arg1—arginase 1; IR—insulin resistance; T2DM—type 2 diabetes mellitus.

Macrophages are distributed throughout various tissues in the body. Tissue-resident macrophages originate from embryonic development and circulating monocytes and are important components of the innate immune system [72]. They recognize and respond to stress signals from damaged organelles, defend against microorganisms and other endogenous and exogenous threats to homeostasis, and serve as the first line of defense in initiating inflammatory responses [73,74]. Macrophages undergo metabolic reprogramming characterized by a marked increase in glycolytic flux, which drives their polarization toward the pro-inflammatory M1 phenotype [75]. A growing body of evidence indicates that macrophage activation status is closely associated with the development and progression of T2DM [76] (Figure 4).

First, hypoxia-inducible factor HIF-1α is a key transcription factor that regulates genes involved in glycolysis and anabolic metabolism. It upregulates the expression of glycolytic enzymes such as GLUT1, hexokinase 2 (HK2), and phosphofructokinase 1 (PFK1), thereby increasing glucose uptake and glycolytic flux to compensate for the impaired energy production of the tricarboxylic acid (TCA) cycle. Second, TCA cycle metabolic blockade leads to accumulation of intermediates such as succinate and fumarate. Succinate, a structural analog of α-ketoglutarate, competitively inhibits prolyl hydroxylase (PHD) activity, blocking HIF-1α prolyl hydroxylation and VHL-mediated degradation. This stabilizes HIF-1α and directly upregulates the expression of pro-inflammatory genes such as IL-1β and NF-κB [77,78]. Third, glycolytically derived metabolites provide substrates for histone acetylation of inflammatory genes, opening the chromatin structure of M1 signature genes such as TNF-α, IL-6, and iNOS, thereby enhancing their transcriptional activity [79]. This metabolic reprogramming causes M1-polarized macrophages to secrete large amounts of pro-inflammatory cytokines such as TNF-α, IL-1β, and IL-6, establishing chronic low-grade metabolic inflammation locally. This process serves as a hub for the transition from intracellular metabolic stress to systemic damage. Inflammatory cytokines act on the liver, muscle, adipose tissue, and pancreatic islets through endocrine and paracrine signaling, directly inducing insulin resistance and β-cell apoptosis as the final effector events.

In addition, in vitro and in vivo studies have shown that anti-inflammatory drugs can block M1 macrophage polarization by inhibiting the PI3K/AKT/mTORC1 signaling pathway and glycolysis [80]. They also effectively suppress lipocalin-2 expression in LPS-stimulated macrophages and reduce NO production and the expression of pro-inflammatory cytokines iNOS and COX-2. They also alleviate neuroinflammation, ROS production, and phagocytosis [81]. Furthermore, they enhance M2 macrophage polarization and the expression of endogenous antioxidants [82]. Together, these findings provide experimental evidence for anti-inflammatory strategies in the treatment of T2DM.

Regarding the role of enhanced glycolysis in macrophages and β-cells, existing evidence presents a time-dependent paradoxical picture. Under short-term high-glucose exposure, increased glycolytic flux can compensate for impaired mitochondrial oxidative phosphorylation and maintain ATP supply to support cell function [83]. However, prolonged high-glucose stimulation leads to abnormal accumulation of glycolytic metabolites. This induces oxidative stress and accelerating cellular dysfunction [77]. This time-dependent difference suggests that enhanced glycolysis may play a dual role in T2DM progression. It may act as an adaptive compensatory mechanism in the early stage and a pathogenic driver of damage in the late stage. Studies focusing on tumor metabolism have shown that the Warburg effect is an adaptive change in early tumorigenesis but becomes an irreversible metabolic phenotype in advanced stages. Whether similar mechanisms occur in metabolic cells in T2DM remains unclear. In addition, different cell types exhibit significant differences in their tolerance to enhanced glycolysis. Macrophages can tolerate higher levels of glycolysis, whereas β-cells are highly sensitive to glycolytic byproducts due to their low expression of antioxidant enzymes.

## 4. Tissue-Specific Heterogeneity of Targeted Insulin Resistance

**Figure 5:**
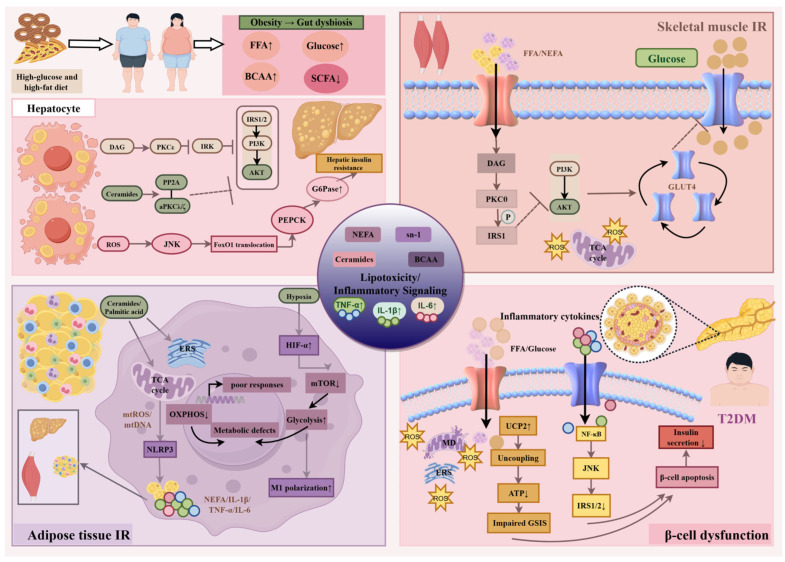
Tissue-specific heterogeneity of targeted insulin resistance. FFA—free fatty acid; NEFA—non-esterified fatty acid; BCAA—branched-chain amino acid; SCFA—short-chain fatty acid; IR—insulin resistance; DAG—diacylglycerol; PKCθ—protein kinase Cθ; IRK—insulin receptor kinase; IRS1/2—insulin receptor substrate 1/2; PI3K—phosphatidylinositol 3-kinase; AKT—protein kinase B; PP2A—protein phosphatase 2A; aPKCλ/ζ—atypical protein kinase C λ/ζ; ROS—reactive oxygen species; JNK—c-Jun N-terminal kinase; FoxO1—forkhead box protein O1; PEPCK—phosphoenolpyruvate carboxykinase; G6Pase—glucose-6-phosphatase; GLUT4—glucose transporter 4; ERS—endoplasmic reticulum stress; TCA cycle—tricarboxylic acid cycle; OXPHOS—oxidative phosphorylation; mtROS—mitochondrial reactive oxygen species; mtDNA—mitochondrial DNA; NLRP3—NLR family pyrin domain-containing 3; HIF-α—hypoxia-inducible factor α; mTOR—mechanistic target of rapamycin; M1—classically activated macrophage; TNF-α—tumor necrosis factor-α; IL-1β—interleukin-1β; IL-6—interleukin-6; UCP2—uncoupling protein 2; MD—mitochondrial dysfunction; ATP—adenosine triphosphate; GSIS—glucose-stimulated insulin secretion; NF-κB—nuclear factor kappa-light-chain-enhancer of activated B cells; T2DM—type 2 diabetes mellitus.

Insulin resistance is a core pathological feature of T2DM, but it exhibits significant heterogeneity across different tissues [84]. The liver, skeletal muscle, and adipose tissue display tissue-specific molecular mechanisms in insulin signaling dysfunction, abnormal glucose and lipid metabolism, and inflammatory responses. In summary, the liver is characterized by excessive gluconeogenesis, skeletal muscle by impaired glucose uptake, adipose tissue by uncontrolled lipolysis and local inflammation, and pancreatic β-cells by impaired glucose-stimulated insulin secretion (GSIS) and apoptosis as the terminal event. The specific mechanisms for each tissue are described below (Figure 5).

### 4.1. Hepatic Steatosis-Driven Insulin Resistance

Under physiological conditions, insulin promotes glucose uptake mediated by glucose transporters in hepatocytes, myocytes, and adipocytes. However, in the setting of hepatic steatosis, the glucose uptake capacity of hepatocytes is impaired, and physiological levels of insulin fail to efficiently reduce blood glucose [85]. Accordingly, disordered hepatic glucolipid metabolism triggers oxidative stress and hepatocellular injury. This further activates Kupffer cells to secrete abundant inflammatory cytokines, ultimately resulting in the development of hepatic insulin resistance and subsequent T2DM progression.

Epidemiological and multi-omics studies have revealed that elevated circulating levels of triglycerides, phosphatidylethanolamine, ceramides, and certain branched-chain amino acids are closely associated with the typical insulin resistance phenotype of T2DM [86,87,88]. During the development of insulin resistance, the concentrations of branched-chain amino acids rise rapidly, accompanied by sustained increases in major unsaturated non-esterified fatty acids (NEFAs). NEFAs and their metabolites act not only as allosteric regulators of hepatic gluconeogenesis but also as inhibitory signaling molecules in peripheral tissues [89,90]. Under a high-glucose and high-fat microenvironment, the liver takes up excessive non-esterified fatty acids (NEFAs), which are then re-esterified to generate sn-1,2-diacylglycerol (DAG). Clinical studies have confirmed that DAG levels are significantly elevated in the hepatocyte membrane of patients with insulin resistance [91]. Accumulation of DAG in the plasma membrane of adipocytes, cardiomyocytes, and hepatocytes activates novel protein kinase C (PKC) isoforms. These isoforms catalyze inhibitory phosphorylation of proximal proteins in the insulin signaling pathway [92]. This insulin signaling defect leads to derepression of the transcription factor FoxO1, which then translocates to the nucleus. There, it transcriptionally upregulates phosphoenolpyruvate carboxykinase (PEPCK) and glucose-6-phosphatase (G6Pase), ultimately driving excessive hepatic gluconeogenesis [93]. In addition to the DAG-PKCε pathway, ceramide inhibits distal insulin signaling through two mechanisms. First, it allosterically activates protein phosphatase 2A (PP2A), promoting dephosphorylation of Akt at Thr308 and Ser473. Second, it stimulates atypical PKC isoforms (aPKCλ and aPKCζ) to prevent Akt from binding to the plasma membrane, thereby blocking signal transduction into the cell.

Elevated levels of free fatty acids (FFAs) in hepatocytes significantly enhance mitochondrial β-oxidation [94]. Chronic overload of this metabolic pathway can lead to fatty acid metabolic imbalance, which in turn induces mitochondrial dysfunction. Incomplete fatty acid oxidation and endoplasmic reticulum stress synergistically increase H_2_O_2_ release, alter cellular redox status, and promote excessive ROS production [95], triggering lipid peroxidation and potentially causing mitochondrial DNA damage [96]. These ROS can inhibit electron transport chain function through redox-sensitive kinases such as ASK1-JNK, further exacerbating insulin signaling dysfunction [8]. Concurrently, hepatocytes overproduce inflammatory cytokines such as TNF-α, IL-1β, and IL-6, which amplify local inflammatory responses through paracrine and autocrine signaling. The synergistic action of these mechanisms disrupts distal insulin signaling pathways, increases hepatic glucose output, and collectively induces insulin resistance.

### 4.2. Skeletal Muscle Insulin Resistance: Core Mechanism of Glucose Uptake Impairment

Skeletal muscle is one of the largest metabolic organs in the body, accounting for approximately 30% of body weight in women and 40% in men. It consists primarily of multinucleated contractile muscle fibers. Skeletal muscle not only plays a key role in movement, physical support, and body temperature regulation but also maintains continuous communication with other tissues through the secretion of various polypeptide factors. It occupies a central position in the regulation of systemic glucose homeostasis [97]. Under insulin stimulation, skeletal muscle takes up approximately 85% of circulating glucose via GLUT4, making it the primary site of postprandial glucose disposal. The core manifestation of skeletal muscle insulin resistance is a marked reduction in insulin-stimulated glucose uptake and glycogen synthesis [98].

After insulin binds to its receptors on the cell membrane, it initiates a signaling cascade involving insulin signaling molecules, including IRS1, PI3K, and Akt. This cascade ultimately promotes GLUT4 translocation from the cytoplasm to the cell membrane to mediate glucose uptake [99]. Multiple pathological mechanisms can impair this process, including accumulation of fatty acid metabolites, inflammatory responses, and mitochondrial dysfunction [100]. Skeletal muscle in T2DM patients often exhibits pathological alterations such as mitochondrial defects and impaired GLUT translocation [101]. A clinical study induced acute muscle insulin resistance by lipid infusion in healthy lean volunteers. Using serial muscle biopsies, the study found that insulin resistance was temporally associated with transient accumulation of diacylglycerol (DAG) in muscle cells, particularly the membrane-associated and cytoplasmic C18:2 DAG species [102]. This accumulation was associated with protein kinase C (PKC) θ activation and increased IRS1-Ser1101 phosphorylation, thereby inhibiting insulin signaling transduction [103]. Impaired insulin signaling triggers a protein phosphorylation cascade, leading to decreased PI3K activity and impaired GLUT4 translocation to the cell membrane. This ultimately reduces cellular glucose uptake [104]. Animal experiments have further supported these mechanisms. In T2DM mice, GLUT4 levels in the gastrocnemius muscle are significantly reduced, and its translocation to the cell membrane is impaired [105]. Similarly, in vitro experiments have demonstrated that in palmitate-induced insulin-resistant myotubes, GLUT4 levels and phosphorylated AMPK levels are markedly decreased, accompanied by reduced glucose uptake capacity [106]. In addition, decreased mitochondrial function in skeletal muscle is closely associated with incomplete fatty acid oxidation. Increased fatty acid flux can lead to mitochondrial dysfunction, which in turn impairs insulin signaling. Mitochondrial dysfunction and lipid accumulation promote each other, forming a vicious cycle. Compared with hepatic insulin resistance, which is characterized by excessive gluconeogenesis, the core defect in skeletal muscle insulin resistance lies in impaired glucose transport and phosphorylation. This defect is a major contributor to the reduction in insulin-stimulated glycogen synthesis and systemic insulin resistance in T2DM patients [107].

Although the role of the DAG-PKCθ pathway in skeletal muscle insulin resistance is relatively well established, its causal relationship has not been consistently verified in human studies. Szendroedi et al. induced acute muscle insulin resistance by lipid infusion and found a close temporal association between transient DAG accumulation and insulin resistance [103]. However, other studies have failed to detect a consistent correlation between DAG levels and insulin sensitivity in muscle biopsies from patients with chronic T2DM [108]. Several factors may contribute to this inconsistency. First, membrane-bound DAG, rather than total DAG, is critical for PKC activation, but most studies have measured only total DAG content. Second, fatty acid chain length may differ. C18:2 DAG may be more pathogenic than other chain lengths, but routine assays do not distinguish between them. Third, there may be heterogeneity among study populations. The pathological mechanisms of acute induced insulin resistance may differ from those of chronic T2DM. Compared with the liver, the evidence base for the DAG-PKC pathway in skeletal muscle is relatively weak. This suggests that DAG-independent mechanisms may also contribute to skeletal muscle insulin resistance.

### 4.3. Adipose Tissue Insulin Resistance: A Key Link in Systemic Insulin Resistance

As a central sensor and signaling hub for whole-body energy status, adipose tissue systematically transmits metabolic signals across the body through multidirectional crosstalk with the liver, skeletal muscle, pancreatic islets and other target organs. Adipose tissue dysfunction is recognized as an essential initiating event in the pathogenesis of insulin resistance and T2DM. Such dysfunction originates from pathological remodeling of the adipose microenvironment, mainly manifested as adipocyte apoptosis and macrophage infiltration, which further reshapes the functional characteristics and secretory profile of adipose tissue. Beyond storing excess energy in the form of lipids, adipose tissue also serves as a critical site for the initiation and regulation of local inflammatory responses. Under obese conditions, the remodeled adipose microenvironment drives macrophage polarization toward the pro-inflammatory M1 phenotype, which secretes abundant inflammatory cytokines, disrupts insulin signaling, and accelerates the progression of insulin resistance. Notably, the immune microenvironment of adipose tissue is highly complex. In addition to macrophages, adipose tissue harbors a variety of immune cells, including T cells (CD4, CD8, Tregs), B cells, natural killer T (NKT) cells, dendritic cells, neutrophils, eosinophils, and mast cells [109]. Experimental studies have further revealed phenotypic switching in adipose tissue macrophages (ATMs). Compared with lean mice, obese mice exhibit a marked increase in macrophage abundance in adipose tissue [110], accompanied by elevated expression of M1-like classical activation-related genes such as TNF-α, Nos2 and Ccr2 [111]. Furthermore, in vitro experiments demonstrate that bone marrow-derived macrophages (BMDMs) exposed to palmitate adopt a phenotype highly similar to ATMs from high-fat diet-fed mice, characterized by robust upregulation of pro-inflammatory cytokines including TNF-α, IL-6 and IL-1β.

Under conditions of nutrient excess, local hypoxia in adipose tissue induces the expression of hypoxia-inducible factor-1α (HIF-1α). Upon activation under hypoxic conditions, HIF-1α reprograms cellular energy metabolism from oxidative phosphorylation (OXPHOS) toward glycolysis. It also directly promotes macrophage polarization toward the pro-inflammatory M1 phenotype. Excessive uptake of free fatty acids by adipose tissue macrophages (ATMs) shifts their metabolic profile away from OXPHOS toward the synthesis of triglycerides, phospholipids and ceramides. This shift triggers intracellular lipotoxicity in macrophages. Fatty acids induce endoplasmic reticulum (ER) structural disruption and ER stress, further leading to mitochondrial calcium overload and dysfunction [112]. Impaired autophagy mediated by ceramides, palmitate and saturated fatty acids results in abnormal accumulation of dysfunctional mitochondria. Meanwhile, lipotoxic substances such as excess fatty acids and ceramides directly impair mitochondrial function in adipocytes and macrophages, triggering the release of mitochondrial reactive oxygen species (mtROS) and mitochondrial DNA (mtDNA) as damage-associated molecular patterns (DAMPs). These mediators activate the NLRP3 inflammasome and further amplify local inflammatory signaling [113]. The synergistic action of these signals drives robust M1 pro-inflammatory macrophage polarization and excessive secretion of TNF-α, IL-6 and IL-1β. This establishes a chronic low-grade inflammatory state within adipose tissue. Such inflammatory signals subsequently spread via the bloodstream to target organs including the liver, skeletal muscle and pancreatic islets [114].

Lipid storage and mobilization in adipose tissue are regulated in a highly coordinated manner, characterized by minute-level regulation and rapid fluctuations in metabolic flux, as typically observed during the fasting–postprandial metabolic transition [115]. Adipose tissue plays a pivotal role in modulating circulating non-esterified fatty acid (NEFA) levels and metabolic flux under both fasting and postprandial conditions. It functions similarly to the liver and skeletal muscle in the regulation of glucose homeostasis and glucose flux [116]. Meanwhile, elevated plasma NEFA levels mainly result from unrestrained lipolysis in insulin-resistant adipose tissue, which constitutes the primary source of systemic lipotoxic burden. Beyond serving as energy substrates, NEFAs also participate in the regulatory mechanism of the glucose–fatty acid cycle [117]. When blood glucose is depleted, NEFAs are utilized as alternative energy fuels; however, ectopic deposition of NEFAs in the liver and skeletal muscle leads to the generation of lipotoxic intermediates such as diacylglycerol (DAG) and ceramides. These metabolites activate PKC and atypical PKC signaling cascades, directly impairing insulin transduction and inducing peripheral insulin resistance [118]. In addition, excessive NEFAs exert direct lipotoxic effects on pancreatic β-cells, exacerbating β-cell injury and accelerating functional exhaustion.

### 4.4. Pancreatic β-Cell Dysfunction

β-cell dysfunction is a key step in the progression from insulin resistance to hyperglycemia in T2DM. Due to the high demand for insulin synthesis and secretion, β-cells are highly sensitive to glucolipid metabolism and endoplasmic reticulum stress. Abnormal cholesterol deposition, human islet amyloid polypeptide accumulation, lipotoxicity, glucotoxicity, and accumulation of inflammatory cytokines can all lead to β-cell dysfunction [119]. Furthermore, β-cell dysfunction can further exacerbate oxidative stress and ER stress. This promotes the progression of inflammatory diseases and the development of T2DM through activation of inflammatory signaling pathways [120]. The molecular mechanisms are described below based on three aspects: lipotoxicity, mitochondrial uncoupling, and inflammatory amplification.

Circulating free fatty acids (FFAs) consist of saturated and unsaturated fatty acids, which are mainly derived from dietary intake, while FFAs synthesized de novo from glucose are predominantly produced in the form of palmitate. Palmitate can be converted into other FFA species through chain elongation and desaturation. Long-term excessive intake of carbohydrates and lipids elevates circulating FFA concentrations. FFAs undergo β-oxidation in mitochondria and peroxisomes, and those with lipotoxic potential preferentially target pancreatic β-cells. Under lipotoxic conditions, lipid droplet deposition in the form of triglycerides is a common phenomenon in multiple mammalian cell types such as hepatocytes and cardiomyocytes, and this pattern also occurs in pancreatic β-cells. Lipotoxicity disturbs cellular metabolism via multiple pathways, including inflammation, autophagy and endoplasmic reticulum stress [121,122].

Insulin receptor substrate (IRS) molecules are key mediators in the insulin signaling pathway and play important roles in fundamental cellular functions such as growth, division, and metabolism [123]. Under lipotoxic conditions, NEFAs activate downstream serine kinases (e.g., JNK, IKKβ, p38, RIP1). These kinases directly phosphorylate inhibitory serine sites on IRS1/2, leading to their degradation. This signaling blockade has two consequences. First, loss of upstream scaffold proteins directly disrupts the PI3K/Akt signaling axis, blocking insulin signal transduction into the cell. Second, IRS1/2 degradation inhibits the binding of transcription factors MafA, PDX1, and NeuroD to the insulin promoter, thereby downregulating insulin gene transcription. This mechanism has been confirmed by in vitro experiments showing that the IRS1/PI3K/Akt/FOXO1 signaling pathway is significantly inhibited in STZ-treated INS-1 cells, leading to increased apoptosis [124].

Under physiological conditions, β-cells highly express glucokinase (GCK) and express only low amounts of lactate dehydrogenase (LDH), ensuring that pyruvate derived from glucose metabolism preferentially enters the mitochondrial oxidative phosphorylation pathway, thereby generating ATP to trigger insulin exocytosis. Under lipotoxic conditions, fatty acids upregulate UCP2 expression, causing uncoupling of oxidative phosphorylation and a marked reduction in ATP production, which in turn impairs glucose-stimulated insulin secretion (GSIS). In vivo experiments have further revealed that IL-1β can induce significant mtDNA release from mitochondria in β-cells via UCP2, exacerbating mitochondrial damage.

In addition, inflammatory signals from adipose tissue and the islets themselves (e.g., HIF-1α, MCP1/CCL2, CXCL1) recruit M1 pro-inflammatory macrophages into the islets. Infiltrating M1 macrophages and damaged β-cells then activate the NLRP3 inflammasome through the TLR4 pathway. The activated NLRP3 inflammasome cleaves caspase-1, releasing large amounts of mature IL-1β and IL-18. These local inflammatory factors directly act back on β-cells, further activating the NF-κB and JNK pathways. This forms a vicious cycle.

## 5. Intervention Strategies Targeting Downstream Glucose Metabolic Pathways

### 5.1. Antioxidant Strategies

Hyperglycemia is the core driver of excessive ROS overproduction and oxidative stress. Abnormal glucose metabolism, such as enhanced glucose uptake and accelerated glycolysis, induces massive ROS generation through multiple mechanisms, including mitochondrial electron transport chain leakage, the hexosamine pathway, and advanced glycation end products (AGEs) formation. Excessive ROS not only damages lipids, proteins and DNA, but also further suppresses oxidative phosphorylation [125]. Therefore, direct ROS scavenging or activation of the endogenous antioxidant system represents one of the key strategies for intervening in downstream glucose metabolic disorders. Clinical trials have demonstrated that hypoglycemic agents such as nuclear factor erythroid 2-related factor 2 (Nrf2) activators, sodium-glucose cotransporter 2 (SGLT2) inhibitors, and glucagon-like peptide-1 receptor agonists (GLP-1RAs) can effectively retard T2DM progression by mitigating oxidative stress. In addition, other antioxidants, including vitamins, lipoic acid, NADPH oxidase (Nox) inhibitors, epigenetic regulators and complement inhibitors, offer promising therapeutic alternatives for T2DM treatment. Accumulating evidence indicates that alleviating oxidative stress reduces the levels of MDA, ROS and JNKs, while upregulating the expression of antioxidant molecules such as Nrf2, SOD, NQO1, HO-1 and GSH-Px, thereby ameliorating the pathological progression of T2DM.

As a first-line therapeutic agent for T2DM, metformin markedly alleviates oxidative stress injury and tissue fibrosis in high-fat-diet/streptozotocin (HFD/STZ)-induced T2DM mice [126]. Its underlying mechanism involves activation of the AMPK/SIRT1-FoxO1 signaling pathway, which effectively relieves oxidative stress and modulates autophagy [127]. Meanwhile, metformin acts as a PGC-1α activator and exerts therapeutic effects against T2DM by maintaining mitochondrial integrity and inhibiting the NLRP3 inflammasome [128]. Beyond hypoglycemic efficacy, sodium-glucose cotransporter 2 (SGLT2) inhibitors also possess definite anti-inflammatory and antioxidant properties [129]. In diabetic rat models, dapagliflozin, either used alone or combined with metformin, improves glycemic control, upregulates organic anion transporter 3 expression, and promotes autophagy [130]. It also suppresses IL-1β secretion in macrophages via the ROS-NLRP3-Caspase-1 signaling cascade [131]. As a member of the insulin hormone family, glucagon-like peptide-1 (GLP-1) enhances insulin secretion and inhibits glucagon release, thereby lowering blood glucose. GLP-1 activates autophagy through regulation of the GLP-1R-AMPK–mTOR–autophagy–ROS axis to ameliorate T2DM pathogenesis. In addition, exenatide treatment effectively reduces macrophage infiltration [132] and alleviates oxidative stress as well as NF-κB activation independent of glycemic changes in diabetic models [133]. Dimethyl fumarate (DMF) exerts immunomodulatory, anti-inflammatory and antioxidant effects by activating Nrf2 and agonizing hydroxycarboxylic acid receptor 2 (HCAR2) [134]. Studies have demonstrated that DMF modulates macrophage subpopulation distribution in mouse skeletal muscle, significantly reduces adipocyte abundance, and inhibits apoptotic and pro-inflammatory pathways, thereby exerting anti-inflammatory, anti-fibrotic and anti-lipotoxic actions [134]. In summary, ROS-mediated tissue damage can be alleviated by upregulating antioxidant enzymes. Therapeutic intervention can also be achieved by reducing ROS production, blocking specific enzymatic reactions, or preventing mitochondrial dysfunction.

Although a large body of basic research has demonstrated that antioxidants can reduce oxidative stress and improve insulin resistance, the results from large-scale clinical trials have been disappointing. Classic antioxidants such as vitamin E, vitamin C, and N-acetylcysteine have failed to consistently show glucose-lowering or complication-reducing effects in T2DM patients [135]. Several factors may explain this discrepancy. First, oxidative stress has a dual role. Appropriate levels of ROS are necessary messengers for insulin signaling, and complete elimination may be counterproductive. Second, the bioavailability of antioxidants is often insufficient. Most oral antioxidants struggle to reach effective concentrations in target tissues. Third, the timing of intervention may be too late. By the time T2DM is diagnosed, oxidative stress may have already reached an irreversible stage. Earlier intervention might be more effective. Fourth, compensatory upregulation may occur. Exogenous antioxidants may inhibit the endogenous Nrf2 system, thereby weakening the body’s own antioxidant reserves. Notably, indirect antioxidant strategies, represented by Nrf2 activators and SGLT2 inhibitors, have shown superior clinical effects compared to direct antioxidants in recent studies [136]. These findings suggest that future antioxidant therapy should shift from directly scavenging ROS toward restoring the endogenous antioxidant defense system and blocking the sources of ROS production.

### 5.2. Repair of Organelle Dysfunction

Increasing attention has been paid to the pivotal roles of mitochondrial dysfunction and endoplasmic reticulum stress in cellular injury and apoptosis during T2DM pathogenesis [137]. In podocytes from STZ-induced diabetic rats, abnormal mitochondrial morphology and reduced MAMs are observed, accompanied by downregulated Mfn2 expression and activation of the unfolded protein response (UPR) pathways, including IRE1, ATF6 and PERK. As a GTPase, Mfn2 mediates outer mitochondrial membrane (OMM) fusion by forming homodimers or heterodimers to bridge adjacent mitochondria [138]. Loss of Mfn2 function markedly impairs mitochondrial fusion efficiency and induces mitochondrial fragmentation. Further studies have shown that Mfn2 is highly enriched in MAMs and regulates multiple biological functions of the endoplasmic reticulum and mitochondria, including calcium homeostasis, protein synthesis, mitochondrial fusion and fission, mitophagy, and inflammatory responses [139,140].

Mfn2 expression is also decreased in the skeletal muscle of T2DM patients. High-glucose (HG)-induced mitochondrial dysfunction, reduced MAMs, and increased apoptosis are accompanied by Mfn2 downregulation and PERK pathway activation. Mechanistically, Mfn2 physically interacts with PERK, and the HG microenvironment weakens their binding affinity [67]. Other studies have shown that the expression of Mfn2 and Mfn1 is markedly reduced, while Fis1 and Drp1 levels are elevated in the renal tissue of diabetic rats. Vitamin D receptor (VDR) agonists can partially restore mitophagy through the Mfn2-MAMs-FUNDC1 axis. They also alleviate mitochondrial swelling, reduce mitochondrial ROS production, and exert beneficial regulatory effects on ERS. Further experiments have confirmed that mitochondrial calcium levels are significantly decreased in HK-2 cells under HG/TGF-β conditions. The decrease may be attributed to impaired MAM integrity and reduced calcium transfer from the endoplasmic reticulum to mitochondria. Treatment with paricalcitol or VDR overexpression plasmid can partially rescue mitochondrial function [141]. These findings directly reveal the intrinsic association among MAM integrity, mitochondrial Ca^2+^ homeostasis, and ATP production.

Early studies have established that the mitochondrial outer membrane voltage-dependent anion channel 1 (VDAC1) physically interacts with endoplasmic reticulum IP3 receptor 1 (IP3R1) via the MAM-associated chaperone GRP75, which directly modulates calcium transfer from the endoplasmic reticulum to mitochondria [142]. Either calcium depletion in the ER or MAM-mediated mitochondrial calcium overload can enhance ROS production and trigger ER dysfunction. This further opens the mitochondrial permeability transition pore (mPTP) and ultimately induces cell death [143]. Recent studies have confirmed that the IP3R1–GRP75–VDAC1 complex mediates the critical crosstalk between ER stress and mitochondrial oxidative stress in the pathogenesis of T2DM. Pharmacological blockade of ERS with the chemical chaperone 4-phenylbutyric acid (4-PBA) [144], or silencing GRP75 via small interfering RNA (siRNA) in HL-1 cardiomyocytes and Hspa9 conditional knockout mouse models, can both restrain ER-to-mitochondria calcium trafficking. This thereby mitigates mitochondrial oxidative stress and calcium overload [145]. In addition, animal experiments demonstrate that tea polyphenol (TP) intervention significantly reduces the protein levels of p-IP3R1 (Ser-1756), GRP75, VDAC1 and MCU in T2DM rats compared with the model group, accompanied by a marked decrease in the proportion of MAM contact length relative to mitochondrial perimeter. These findings indicate that TP ameliorates memory impairment in T2DM rats by regulating GRP75-targeted MAM homeostasis in hippocampal neurons. This provides a novel mechanistic insight into the neuroprotective effects of TP in T2DM-associated complications [146].

### 5.3. Anti-Systemic Inflammation Strategies

Hyperglycemia-induced abnormal glucose metabolism serves not only as the primary source of oxidative stress but also provides the metabolic foundation for the chronic inflammatory cascade. Enhanced glycolysis, activation of the hexosamine biosynthetic pathway (HBP), and reprogramming of the pentose phosphate pathway (PPP) can directly or indirectly regulate NLRP3 inflammasome activation, macrophage polarization, and pro-inflammatory cytokine release through the accumulation of metabolic intermediates and modification of signaling molecules [147]. Among these events, abnormally elevated glycolytic flux represents a hallmark metabolic reprogramming feature of inflammatory cells, particularly M1 macrophages. Such metabolic reprogramming occurs dynamically at different stages of inflammation and is precisely modulated by the type, abundance, and competitive interaction of regulatory factors such as IL-4 and IFN-γ in the local microenvironment. Furthermore, hypoxia and lactate accumulation within the microenvironment can independently trigger metabolic reprogramming in macrophages [148].

Pharmacological intervention targeting glucose metabolic reprogramming has demonstrated cardiorenal protective effects beyond simple hypoglycemia. Sodium-glucose cotransporter 2 inhibitors (SGLT2is) suppress sympathetic overactivation, a common complication in T2DM patients, by alleviating oxidative stress and inflammation [149]. A meta-analysis covering six major cardiovascular and renal outcome trials in T2DM confirmed that SGLT2is reduced the composite renal endpoint by 36%, independent of baseline atherosclerotic cardiovascular disease status [150]. Mechanistic studies have shown that empagliflozin ameliorates endothelial dysfunction and arterial stiffness in diabetic rats and delays the progression of nephropathy [151]. Moreover, empagliflozin reduces adipose tissue mass in normoglycemic animals and lowers blood pressure in diabetic patients [152,153]. Dapagliflozin has also been shown to significantly decrease the risk of renal events in cardiorenal protection trials [154]. Glucagon-like peptide-1 receptor agonists (GLP-1 RAs) improve glycemic control independent of insulin signaling by enhancing endothelial function, optimizing body weight and blood pressure regulation, and exerting direct anti-inflammatory and anti-fibrotic effects on renal tissues. Combined administration of SGLT2is and GLP-1 RAs achieves augmented protection against cardiovascular and renal deterioration through synergistic modulation of hemodynamic, metabolic, and inflammatory pathways [155].

Beyond the above broad intervention strategies, precise targeting of key rate-limiting enzymes involved in glycolysis has gradually become a cutting-edge research direction for T2DM therapy. As a pivotal rate-limiting enzyme of glycolysis, upregulated PFKFB3 expression enhances glycolytic activity in endothelial cells and macrophages. It also promotes the production of pro-inflammatory cytokines such as IL-1β and TNF-α. Pharmacological inhibition of PFKFB3 markedly reduces glycolytic flux and alleviates vascular inflammation in diabetic atherosclerosis models [156,157]. Accumulating evidence indicates that PFKFB3 mediates diabetic organ damage by regulating immune inflammatory responses, fibrosis, vascular dysfunction, as well as pancreatic β-cell function and survival. This suggests that PFKFB3 represents a promising therapeutic target for diabetic complications [157]. As a functionally complementary molecule, PKM2 acts as a core linker between glycolysis and inflammatory signaling. In addition to catalyzing the conversion of phosphoenolpyruvate to pyruvate, PKM2 can translocate into the nucleus and cooperate with HIF-1α to initiate the transcription of NLRP3 inflammasome-related genes. Studies have verified that PKM2 activators can reverse LPS-induced M1 macrophage polarization and suppress NLRP3-dependent caspase-1 activation as well as IL-1β secretion [158]. Notably, elevated levels of succinate stabilize HIF-1α to sustain the M1 macrophage phenotype, which is closely correlated with exacerbated inflammatory responses [83]. In the context of diabetic encephalopathy, the glycolytic status of central microglia determines their inflammatory phenotype. A high-glucose microenvironment drives a metabolic shift from oxidative phosphorylation to glycolysis in microglia, accompanied by PKM2 nuclear translocation and NLRP3 inflammasome activation. This process triggers the release of IL-1β and TNF-α, ultimately leading to neuronal injury and cognitive dysfunction [159]. Previous studies have demonstrated that citrus-derived polyphenols, including hesperidin, naringenin, and nobiletin, can systematically remodel the glucose metabolic network via multi-target regulation of the AMPK, PI3K/Akt, NF-κB and Nrf2 signaling pathways. This thereby ameliorates insulin resistance and metabolic inflammation [160].

### 5.4. Traditional Chinese Medicine and Natural Products

In recent years, in addition to continuous breakthroughs in Western medicine represented by SGLT2 inhibitors and GLP-1 receptor agonists, bioactive components derived from traditional Chinese medicine (TCM) have exhibited unique advantages in regulating macrophage metabolic reprogramming and immune homeostasis. Compared with synthetic chemical drugs, which are often limited to single-target action or accompanied by potential adverse reactions, these natural products exert direct antioxidant and anti-inflammatory effects. More importantly, they can remodel macrophage metabolism through epigenetic regulation and modification of metabolic enzyme activity, thereby ameliorating insulin resistance and chronic inflammation in T2DM.

Berberine induces aerobic glycolysis by blocking the tricarboxylic acid cycle and modulates cytokine responses in bone marrow-derived macrophages (BMDMs) isolated from mouse and human peripheral blood mononuclear cells (PBMCs). Berberine gradually upregulates GLUT1 expression and enhances total cellular hexokinase activity in BMDMs. Moreover, in LPS-injected mice, berberine treatment attenuates the elevation of serum TNF-α and reductions in blood glucose [161]. Puerarin activates the AMPK-mTOR and PPARγ-NF-κB signaling pathways, modulates insulin signaling and glycolipid metabolism, and alleviates inflammatory injury [162]. Meanwhile, puerarin reduces macrophage infiltration in adipose tissue, downregulates TNF-α expression, and improves obesity-associated inflammation and dyslipidemia [163]. Resveratrol is a polyphenolic compound that is widely present in various plants and synthesized mainly under pathogen-induced stress. It possesses multiple biological activities, including antioxidant, anti-inflammatory, anti-carcinogenic, anti-aging, anti-diabetic and neuroprotective effects [164]. Under high-glucose conditions, resveratrol markedly inhibits NO production and pro-inflammatory interleukin secretion in LPS-stimulated macrophages. Existing evidence indicates that resveratrol effectively suppresses the release of pro-inflammatory cytokines from macrophages and remodels the extracellular metabolic microenvironment under hyperglycemic conditions [165]. In vitro, astragalus polysaccharides inhibit the differentiation of LPS/HG-stimulated THP-1 cells into pro-inflammatory M1 macrophages. This is accompanied by reduced ROS generation and decreased secretion of pro-inflammatory cytokines. Meanwhile, astragalus polysaccharides promote M2 polarization and the release of anti-inflammatory factors (IL-4, IL-10, and Arg-1) by activating the Nrf2/HO-1 signaling pathway in THP-1-derived macrophages. Such regulatory effects can be abolished by Nrf2 siRNA interference. In addition, astragalus polysaccharides enhance endothelial migration and angiogenesis in the co-culture system of HUVECs and macrophages under high-glucose conditions [166]. Collectively, these studies suggest that natural products can regulate macrophage glycolysis and polarization through multi-target mechanisms, providing novel therapeutic strategies for T2DM. Nevertheless, current research on TCM interventions targeting the upstream event of macrophage metabolic reprogramming remains relatively limited. Further in-depth investigations are warranted in the future.

Although active ingredients in traditional Chinese medicine exhibit unique advantages in regulating macrophage metabolic reprogramming, the quality of evidence from existing studies requires careful evaluation. The following major limitations currently exist. First, there is heterogeneity in study design. Different studies use different cell models, drug concentrations, and intervention durations, making it difficult to directly compare results or perform reproducible validation. Second, there is overinterpretation of mechanisms. Most studies infer targets by detecting phosphorylation levels of pathway proteins but lack target validation experiments. Whether natural products directly bind to target proteins remains unclear. Third, there is a lack of pharmacokinetic evidence. Most studies use drug-containing serum for administration but do not measure the actual concentrations of active ingredients in the serum. They also do not determine whether effective concentrations can be reached in target tissues. Notably, research on berberine is relatively advanced, with multiple target validation and PK/PD studies supporting its effects [161]. In contrast, the evidence base for other natural products remains relatively weak.

## 6. Conclusions

In summary, glucose metabolic reprogramming plays an important role in the initiation and progression of T2DM. The hyperglycemic microenvironment drives metabolic cells such as macrophages to shift their energy metabolism from oxidative phosphorylation to aerobic glycolysis. This metabolic transition not only meets the energy demands for rapid cell proliferation and functional activation but also initiates and amplifies downstream pathological cascades through the accumulation of metabolic intermediates and the modification of signaling molecules. As an important structure in this pathological process, oxidative stress triggers excessive ROS production via multiple pathways, including mitochondrial electron transport chain leakage, hexosamine pathway activation, and advanced glycation end product (AGEs) formation. Mitochondrial–endoplasmic reticulum dysfunction further exacerbates cellular injury and apoptosis through the disruption of mitochondria-associated endoplasmic reticulum membrane (MAM) integrity, calcium homeostasis imbalance, and abnormal activation of the unfolded protein response (UPR). The synergistic interplay of these downstream effectors ultimately leads to pancreatic β-cell dysfunction, insulin resistance and chronic inflammation, thereby facilitating the progression of T2DM and its related complications.

Although there is wide recognition of the key role of glucose metabolic reprogramming and its downstream mechanisms in T2DM, the following limitations still exist. First, the regulatory mechanism of MAMs in T2DM remains incompletely understood. MAMs serve as critical sites for communication between mitochondria and the endoplasmic reticulum, playing core roles in calcium signaling, lipid metabolism, mitophagy, and inflammatory responses. Notably, inconsistencies in the direction of MAM changes across different studies may be related to disease stage, tissue type, and detection methods. In early T2DM, MAMs may increase compensatorily to maintain organelle communication, whereas in late stages, MAM structural disruption becomes more severe. Furthermore, the liver and pancreatic β-cells differ in their sensitivity to MAM changes. β-cells, due to their high demand for insulin secretion, are more dependent on MAM-mediated calcium transport. Currently, there is a lack of molecular markers to distinguish between physiological and pathological MAM increase. There are also a lack of technical approaches for real-time monitoring of MAM dynamics. Second, evidence for mitochondrial fragmentation largely comes from cross-sectional studies, making it difficult to establish temporal relationships. Future studies need to clarify causal direction using conditional gene knockout animal models and live cell dynamic imaging techniques. In addition, the functional significance of mitochondrial fragmentation may differ across tissues, but systematic cross-tissue comparative studies are currently lacking. Third, clinical translational research is insufficient. Existing evidence is derived primarily from cell and animal studies. There are a lack of large-scale, multicenter clinical cohort studies that validate the effects of interventions targeting glucose metabolic reprogramming or downstream stress pathways.

Future research may proceed in the following directions. First, targeting mitochondria–endoplasmic reticulum interactions represents a new therapeutic approach for T2DM. Future studies should focus on establishing quantitative assessment systems for MAM function, clarifying the patterns of MAM changes at different disease stages, and developing MAM-targeted intervention strategies. Secondly, the prediabetic stage serves as a critical window for T2DM prevention. At this stage, abnormal glucose metabolism has not yet become irreversible, and both oxidative and endoplasmic reticulum stress remain reversible. Early intervention targeting downstream effectors of glucose metabolic reprogramming may block or delay the progression from prediabetes to overt T2DM. Finally, integrating metabolomics, transcriptomics, proteomics and single-cell sequencing technologies can systematically reveal the dynamic characteristics of glucose metabolic reprogramming across different T2DM stages, as well as its crosstalk with downstream stress signaling pathways. The anticipated findings will provide novel therapeutic targets and theoretical evidence for the early prevention and clinical management of T2DM.

## Data Availability

No new data were created or analyzed in this study. Data sharing is not applicable to this article.

## Abbreviations

The following abbreviations are used in this manuscript:

ACLY	ATP-citrate lyase.
AKT	Protein kinase B.
AMPK	Adenosine monophosphate-activated protein kinase.
Arg-1	Arginase 1.
ATF4	Activating transcription factor 4.
ATF6	Activating transcription factor 6.
Bax	Bcl-2-associated X protein.
Bcl-2	B-cell lymphoma 2.
BCAA	Branched-chain amino acid.
CHOP	C/EBP homologous protein.
Cyt c	Cytochrome c.
DAG	Diacylglycerol.
DAMPs	Damage-associated molecular patterns.
Drp1	Dynamin-related protein 1.
EGF/VEGF	Epidermal growth factor/vascular endothelial growth factor.
eIF2α-P	Phosphorylated eukaryotic initiation factor 2α.
ERS	Endoplasmic reticulum stress.
ETC	Electron transport chain.
FFA/NEFA	Free fatty acid/non-esterified fatty acid.
FoxO1	Forkhead box protein O1.
G6Pase	Glucose-6-phosphatase.
GLUT1	Glucose transporter 1.
GLUT4	Glucose transporter 4.
GLUTs	Glucose transporters.
GPx-1	Glutathione peroxidase 1.
GRP75	Glucose-regulated protein 75.
GSH-Px	Glutathione peroxidase.
HG	High glucose.
HIF-1α	Hypoxia-inducible factor 1-alpha.
HK2	Hexokinase 2.
HO-1	Heme oxygenase-1.
IL-1β	Interleukin-1β.
IL-6	Interleukin-6.
iNOS	Inducible nitric oxide synthase.
IP3R	Inositol 1,4,5-trisphosphate receptor.
IRE1α	Inositol-requiring enzyme 1α.
IRK	Insulin receptor kinase.
IRS1/2	Insulin receptor substrate 1/2.
JNK	c-Jun N-terminal kinases.
M1	Classically activated macrophage.
M2	Alternatively activated macrophage.
MAPK	Mitogen-activated protein kinase.
MAMs	Mitochondria-associated endoplasmic reticulum membranes.
MDA	Malondialdehyde.
MFN1/2	Mitofusin 1/2.
mPTP	Mitochondrial permeability transition pore.
mtDNA	Mitochondrial DNA.
mtROS	Mitochondrial reactive oxygen species.
mTOR	Mechanistic target of rapamycin.
NF-κB	Nuclear factor kappa-light-chain-enhancer of activated B cells.
NLRP3	NLR family pyrin domain-containing 3.
NQO1	NADPH quinone oxidoreductase.
Nrf2	Nuclear factor erythroid 2-related factor 2.
NRF1/2	Nuclear respiratory factor 1/2.
OXPHOS	Oxidative phosphorylation.
p38	p38 mitogen-activated protein kinase.
PEPCK	Phosphoenolpyruvate carboxykinase.
PFK1	Phosphofructokinase 1.
PGC-1α	Peroxisome proliferator-activated receptor gamma coactivator 1-alpha.
PHD	Prolyl hydroxylase.
PI3K	Phosphatidylinositol 3-kinase.
PKCθ	Protein kinase C θ
PP2A	Protein phosphatase 2A.
ROS	Reactive oxygen species.
SCFA	Short-chain fatty acid.
SERCA	Sarco/endoplasmic reticulum calcium ATPase.
SIRT1	Sirtuin 1.
SOD	Superoxide dismutase.
SOD-1	Superoxide dismutase 1.
T2DM	Type 2 diabetes mellitus.
TCA cycle	Tricarboxylic acid cycle.
TFAM	Mitochondrial transcription factor A.
TLR4	Toll-like receptor 4.
TNF-α	Tumor necrosis factor-α.
TRX	Thioredoxin.
UCP2	Uncoupling protein 2.
VDAC	Voltage-dependent anion channel.
XBP1	X-box binding protein 1.
XBP1s	Spliced X-box binding protein 1.
ΔΨm	Mitochondrial membrane potential.
